# Structure-Guided Discovery and Biochemical Validation of Novel Small-Molecule Inhibitors Predicted to Target the CCHFV OTU Protease Y89-W99 Pocket

**DOI:** 10.3390/ijms27135661

**Published:** 2026-06-23

**Authors:** Sezer Akgöl, Fatih Kocabaş

**Affiliations:** 1 Medizinische Klinik und Poliklinik I, LMU University Hospital, 80336 Munich, Germany; sezer.akoel@med.uni-muenchen.de; 2 Department of Molecular Biology and Genetics, Faculty of Engineering and Natural Sciences, Istanbul Atlas University, 34403 Istanbul, Türkiye

**Keywords:** CCHFV, OTU protease, deubiquitinase, small-molecule inhibitors, structure-based design, antiviral discovery

## Abstract

Crimean–Congo hemorrhagic fever virus (CCHFV) remains a major public health threat due to its high mortality rates and the absence of approved antiviral therapies. The viral ovarian tumor (OTU) protease is a critical virulence factor that suppresses host innate immunity through its deubiquitinase activity, making it an attractive therapeutic target. In this study, we employed a structure-guided approach to identify and validate novel small-molecule inhibitors targeting the non-catalytic Y89-W99 pocket of the OTU protease. Recombinant OTU protease was successfully expressed, purified, and refolded, yielding a soluble and enzymatically active protein. Cellular assays confirmed that the enzyme retains robust deubiquitinase activity, significantly reducing global ubiquitin conjugates in mammalian cells. In silico analysis of a putative DUB inhibitor library identified several candidate inhibitors with favorable binding interactions within the Y89-W99 pocket. Biochemical validation using a fluorometric Ub-AMC assay revealed that multiple small molecules strongly inhibit OTU activity, including OTUi-10 (~93% inhibition), OTUi-13 (~87%), OTUi-1 (~85%), OTUi-4 and OTUi-11 (~81%), and OTUi-9 (~76%). Additional moderate inhibitors included OTUi-12 (~67%), OTUi-19 and OTUi-21 (~66%), and OTUi-5 (~57%). In silico drug-likeness and toxicity profiling filtered the library to four fully compliant candidates, OTUi-4, OTUi-10, OTUi-11, and OTUi-12, all free of predicted toxicity alerts. These findings suggest that the Y89–W99 pocket may be a pharmacologically relevant site worthy of further investigation and identify OTUi-10, OTUi-4, and OTUi-11 as promising preliminary hit compounds. The results also provide initial insights that may guide future optimization and mechanistic studies of OTU protease inhibitors targeting CCHFV.

## 1. Introduction

Crimean–Congo hemorrhagic fever virus (CCHFV) is a tick-borne negative-strand RNA virus of the family Nairoviridae that causes a severe hemorrhagic syndrome with case-fatality rates reaching 30–50% in hospitalized patients [1,2]. The virus is endemic across Africa, Asia, the Middle East and southeastern Europe, and its geographic range is expanding through the wide dispersion of Hyalomma ticks, including those carried by migratory birds [3,4,5,6]. CCHF is therefore recognized as a significant public health threat and a Category C potential bioterrorism agent. Despite its severity, no licensed vaccine or specific antiviral therapy is currently available; the nucleoside analog ribavirin is used off-label but shows limited efficacy in controlled studies [7,8,9]. This unmet medical need has prompted the World Health Organization to list CCHF as a priority disease for research and development of countermeasures.

A key determinant of CCHFV virulence is the ovarian tumor (OTU) protease domain, which resides in the N-terminal region of the viral L protein [10]. The OTU protease possesses dual deubiquitinase (DUB) and deISGylase activities, enabling it to cleave both ubiquitin (Ub) and the interferon-stimulated gene 15 (ISG15) protein from host targets [11,12]. Structural studies revealed that the CCHFV OTU recognizes the conserved C-terminal “LRLRGG” motif shared by Ub and ISG15, with the catalytic cysteine (C40) and histidine (H151) forming the active site that mediates hydrolysis [12,13]. By stripping Ub and ISG15 from signaling intermediates, the OTU protease blunts the activation of NF-κB and interferon-regulatory factors, thereby suppressing the production of type I interferons and pro-inflammatory cytokines [14,15]. Reverse-genetics studies demonstrated that OTU DUB activity is essential for viral evasion of the innate immune response, whereas the deISGylase activity reverses ISG15-mediated antiviral effects [15]. Furthermore, the OTU domain is required for efficient viral RNA polymerase function through a mechanism that appears to involve regulation by ISG15 [16]. Collectively, these findings establish the OTU protease as both a critical virulence factor and a high-value drug target.

The therapeutic potential of inhibiting the CCHFV OTU has spurred several drug-discovery efforts. The first reported small-molecule inhibitors, homidium bromide and phenanthrenequinone, were identified through docking studies and showed dose-dependent inhibition in a Ub-AMC fluorogenic assay [17]. A subsequent study described a 2-aminothiazole series optimized to a competitive inhibitor (IC_50_ = 10.7 μM) via a Ub-rhodamine-110 assay; however, the compound also inhibited several human DUBs (USP7, UCHL5, OTUD1, OTUD7B), revealing a selectivity liability that limits further development [18]. In parallel, engineered ubiquitin variants (UbVs) proved to be potent and selective protein-based inhibitors of the CCHFV OTU. The ubiquitin variant CC4 (UbV-CC4) binds the OTU with high affinity and blocks viral replication in cell culture, although its mechanism appears to involve interference with RNA synthesis rather than rescue of the immune response, and in vivo delivery via an adenoviral vector failed to protect mice from lethal CCHFV challenge [19,20]. Computational drug-repurposing campaigns have nominated candidates such as paromomycin from FDA-approved libraries, but these leads await experimental validation [21]. Despite these advances, no small-molecule OTU inhibitor has yet demonstrated cell-based antiviral activity, and no compound has advanced to animal efficacy studies.

Our own prior work has contributed critical structural, functional, and translational insights to the CCHFV OTU inhibitor field. Through phylogenetic and homology analysis of more than fifty viral and non-viral OTU-related proteases, we identified a previously unrecognized conserved pocket formed by residues Y89-W99 [22]. Docking of a set of in vitro-validated inhibitors predicted that these small molecules preferentially bind to the Y89-W99 pocket rather than to the canonical catalytic triad (D37-C40-H151), defining a new pharmaceutical targeting site [22]. Using this pocket as a search space, we conducted an in silico screen of >600,000 PubChem compounds and identified over 300 hits with enhanced predicted binding affinities (up to −11.0 kcal/mol), including small molecules NSC658721 and NSC683337 [22]. Notably, structural clustering showed that 75% of the most potent, non-cytotoxic candidates share common chemical substructures. To support biochemical validation of such hits, we developed a reproducible fluorometric Ub-AMC assay that enables sensitive detection of OTU activity [17]. Together, these findings provided a robust platform for rational, structure-guided development of OTU protease inhibitors.

Several key challenges remained in the pursuit of clinically useful CCHFV OTU inhibitors. First, the shallow and extended substrate-binding surface of the OTU makes it inherently difficult to develop small molecules with adequate potency and selectivity. The most advanced small-molecule inhibitor to date is non-selective, raising concerns about off-target effects on host DUBs [18]. Second, published inhibitors have only been validated in biochemical assays; there is a conspicuous absence of cell-based antiviral data and in vivo efficacy. Third, the structural biology of the OTU in complex with small-molecule ligands is limited to docking models, and no co-crystal structure of a drug-like inhibitor bound to the OTU has been reported, hindering structure-based optimization. Fourth, the physiological role of the OTU in the context of the full-length L protein and authentic infection remains incompletely understood, complicating the selection of the most translationally relevant assay readouts [16].

A recent study demonstrated that Plasmodium parasites, including *P. falciparum*, *P. vivax*, and *P. yoelii*, encode highly conserved viral OTU-like proteases with ubiquitin and ISG15 deconjugation activities [23]. Structural modeling revealed that the catalytic and inhibition pockets of these malarial OTU proteins share conserved residues with viral OTU domains. A targeted small-molecule screen identified two compounds that broadly inhibit Plasmodium OTU proteases, achieving IC_50_ values as low as 30 nM in biochemical assays and demonstrating potent anti-malarial activity with IC_50_ values of 4.1–6.5 µM against cultured parasites. The authors further characterized these inhibitors through enzyme kinetics, drug-likeness profiling, ADME prediction, QSAR modeling, and molecular dynamics simulations. Importantly, this work also provided a curated library of putative DUB/OTU inhibitor compounds, which served as the focused screening set tested against CCHFV OTU in the present study.

Given the urgent need for specific CCHF antivirals and the validated tractability of the OTU protease as a target, the development of next-generation inhibitors that address the selectivity, potency, and pharmacokinetic deficiencies of current leads is imperative. Our previous identification of the Y89-W99 pocket and the common chemical scaffolds that are predicted to occupy it provides a unique starting point for this endeavor. In the present manuscript, we describe the structure-guided identification of these scaffolds, their in vitro and cellular characterization, and the evaluation of their antiviral efficacy in relevant infection models, with the overarching goal of informing future drug discovery efforts.

## 2. Results

### 2.1. Recombinant Expression and Purification of CCHFV OTU Protease

**Figure 1:**
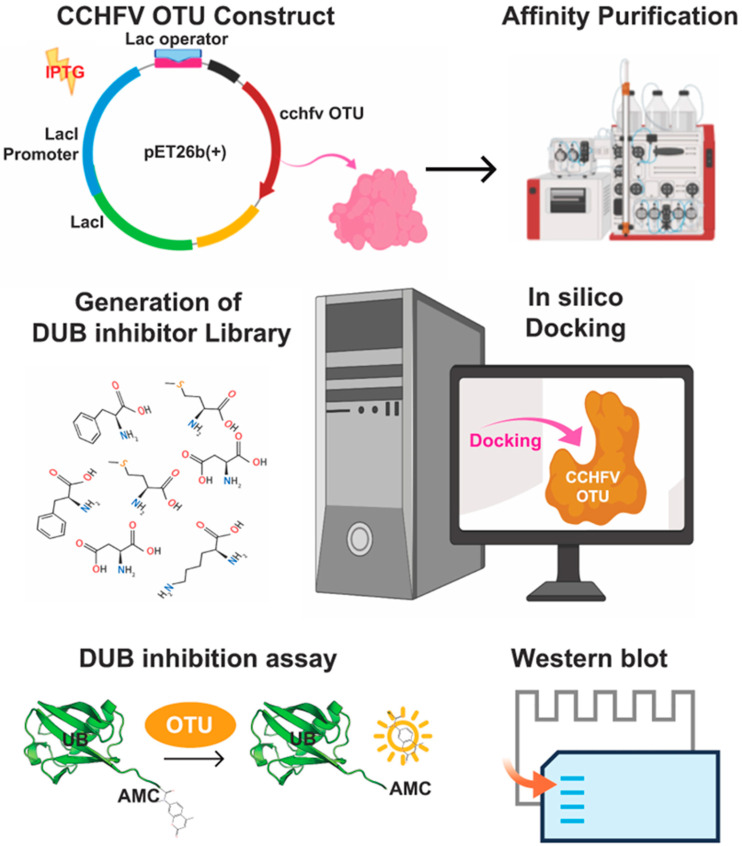
Schematic of the integrated research strategy for CCHFV OTU inhibitor development. The workflow integrates four major stages: (i) bacterial expression and refolding of recombinant CCHFV OTU protease to obtain active enzyme; (ii) establishment of a fluorometric deubiquitinase (DUB) assay (Ub-AMC) to quantify OTU activity; (iii) in silico structure-based analysis of putative DUB inhibitors against the conserved Y89-W99 pocket to prioritize candidate inhibitors; and (iv) in vitro validation of hits through single-concentration (20 μM) DUB activity screening assays (Ub-AMC).

**Figure 2:**
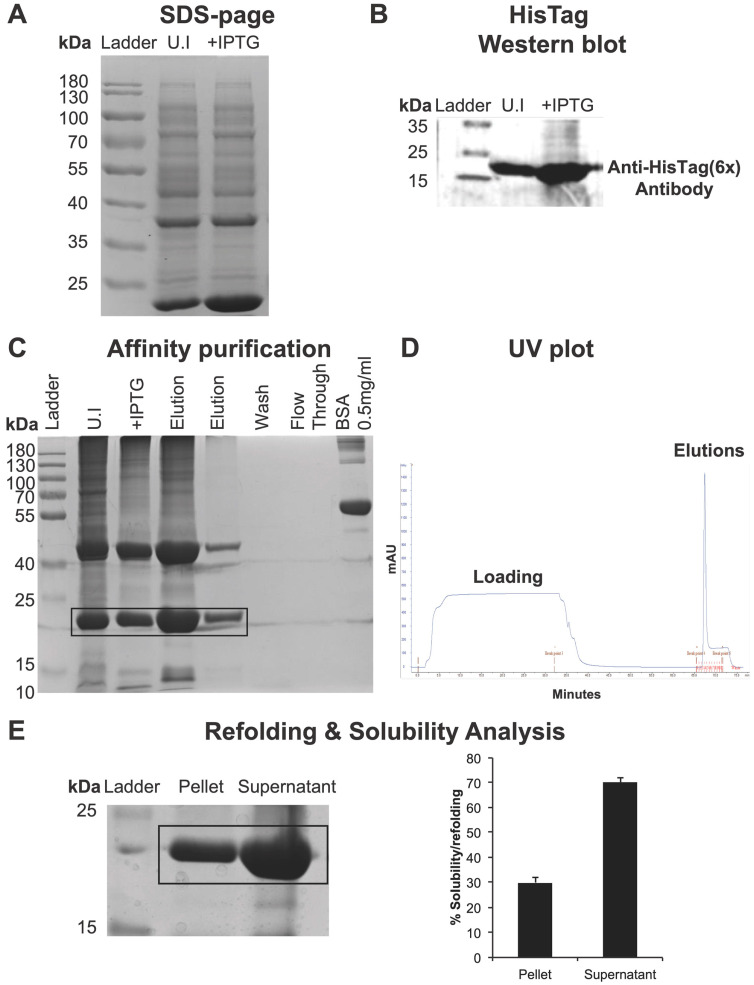
Production and refolding of recombinant CCHFV OTU protease. (**A**) SDS-PAGE analysis of total *E. coli* BL21(DE3) lysates expressing C-terminal His-tagged CCHFV OTU before (uninduced) and after 1 mM IPTG induction. The protein migrates at the expected molecular mass of 21.7 kDa. (**B**) Anti-His Western blot confirming specific expression of His-tagged OTU. (**C**) SDS-PAGE of flow-through (FT), wash (W), and elution fractions demonstrating high purity in the main fraction. (**D**) Representative chromatogram of affinity purification. The sharp peak corresponds to bound OTU. (**E**) (**Left**) Refolding and solubility analysis of OTU in PBS supernatant and pellet; (**Right**) its quantification assessed by SDS-PAGE. *n* = 3.

To enable biochemical and cellular studies, a C-terminally His-tagged CCHFV OTU protease was expressed in *E. coli* BL21(DE3) (Figure 1). Induction with 1 mM IPTG at 25 °C yielded a prominent band migrating at the expected molecular mass of 21.7 kDa, visible by SDS-PAGE (Figure 2A). The identity of the overexpressed protein was confirmed by anti-His Western blotting (Figure 2B). The recombinant viral OTU (vOTU) was purified under denaturing conditions via immobilized metal-affinity chromatography. A single sharp elution peak was observed in the chromatogram, and SDS-PAGE analysis of the main elution fraction showed high purity (Figure 2C,D). The pellet and supernatant fractions were analyzed by SDS-PAGE (Figure 2E, left). Protein solubility was quantified by comparing the band intensity of the supernatant to that of the total protein (Figure 2E, right). Under PBS conditions, vOTU exhibited >70% solubility (Figure 2E). These results establish a robust production pipeline that yields highly pure, soluble viral OTU proteases amenable to high-throughput screening.

### 2.2. Cellular Deubiquitinase Activity of CCHFV OTU

**Figure 3:**
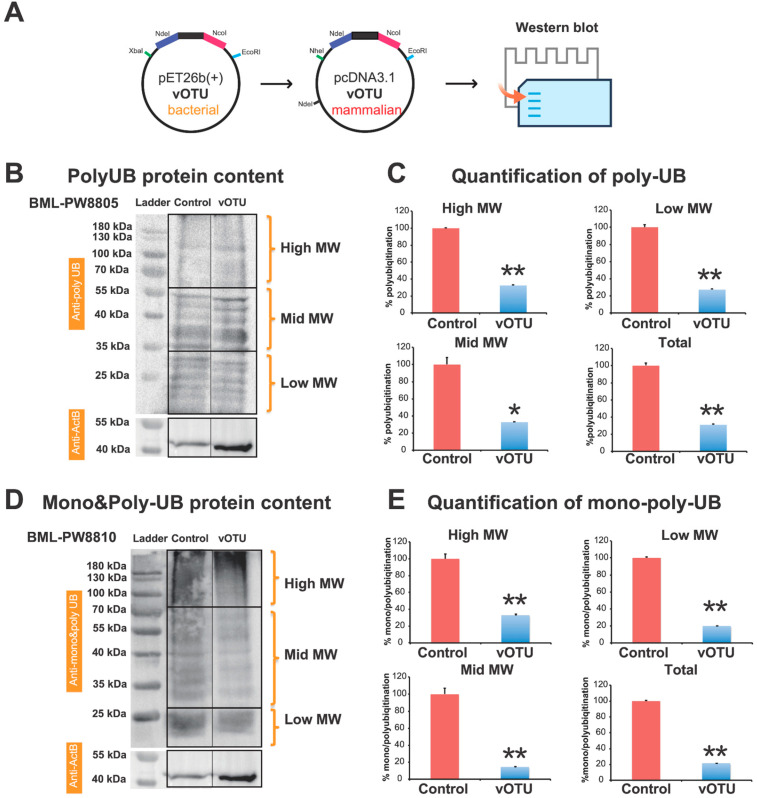
Cellular deubiquitinase activity of CCHFV OTU protein. (**A**) Cloning CCHFV OTU (vOTU) from pET-26b(+) vector into pcDNA3.1 expression vector followed by testing in Western blot analysis of ubiquitome in mammalian cells. (**B**) HEK293T cells were transfected with CCHFV OTU-pcDNA3.1 vector or empty vector (pcDNA3.1). Whole-cell lysates were immunoblotted for poly-ubiquitin conjugates. OTU-expressing cells show a marked decrease in high-molecular-weight ubiquitinated species compared to the control, indicating active deubiquitination. (**C**) Densitometric quantification of total ubiquitin-conjugate signal (normalized to β-actin). (**D**) Analysis mono-poly ubiquitin-conjugated proteins in whole-cell lyses. (**E**) Densitometric quantification of mono-poly ubiquitin-conjugated protein signal (normalized to β-actin). Statistical significance determined by two-tailed Student’s *t*-test (*n* = 3, * *p* < 0.05, ** *p* < 0.01).

To verify that the recombinant enzyme retains activity in a cellular context, the OTU coding sequence was subcloned into a mammalian expression vector and transfected into HEK293T cells. Immunoblotting of whole-cell lysates with an anti-polyubiquitin antibody revealed that OTU expression markedly reduced the abundance of poly-ubiquitin conjugates compared to cells transfected with an empty vector (Figure 3B). Densitometric quantification of the high, mid, low or total ubiquitin-conjugate signal, normalized to β-actin, confirmed a statistically significant decrease (Figure 3C). Further analysis of mono- and polyubiquitinated proteins showed a consistent reduction in the viral OTU-expressing samples (Figure 3D), with a significant drop in the global ubiquitinated protein level (Figure 3E; *** *p* < 0.001). These findings demonstrate that the bacterially expressed and refolded CCHFV OTU is a fully functional deubiquitinase in mammalian cells, capable of broadly stripping ubiquitin from host substrates, and support its role in dampening ubiquitin-dependent innate immune signaling.

### 2.3. In Silico Identification and Characterization of Novel OTU Inhibitors Targeting the Y89-W99 Pocket

**Figure 4:**
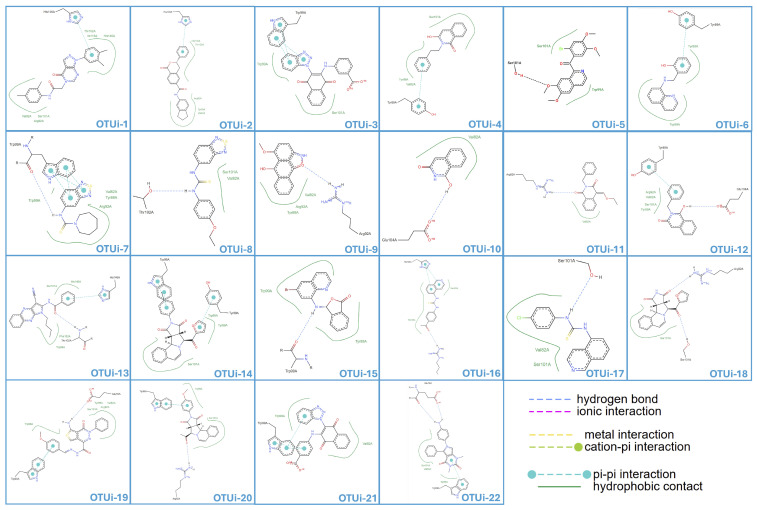
In silico identification and interaction profiling of small-molecule OTU inhibitors. The crystal structure of the CCHFV OTU domain (PDB ID: 3PRP) was prepared, and a docking grid was defined around the Y89–W99 binding pocket. A library of putative DUB inhibitors was screened using AutoDock Vina. The 2D interaction maps of the top-ranked compounds, designated OTUi-1 through OTUi-22, illustrate their binding modes within the OTU active site. Key interactions, including hydrogen bonds, ionic interactions, metal coordination, cation–π interactions, π–π stacking, and hydrophobic contacts, are indicated.

With active protease in hand, we pursued a structure-guided virtual screening approach to identify small-molecule inhibitors. The crystal structure of CCHFV OTU (PDB: 3PRP) was used to define a docking grid centered on the previously identified conserved Y89-W99 pocket. A selection of putative DUB inhibitor library of small molecules was docked using AutoDock Vina, and molecules were inspected for their predicted binding poses and intermolecular contacts (see the list of compounds at [23]). The library includes diverse chemotypes: phthalimides (unsubstituted and N-alkylated), thioureas, benzothiadiazoles, naphthoquinone–benzotriazole hybrids, and polycyclic aromatics. Compounds were selected based on structural diversity and predicted binding properties from prior computational enrichment analyses. The two-dimensional interaction map of representative hits illustrates hydrogen-bond, hydrophobic, and π-stacking interactions with key residues lining the pocket, including Y89 and W99 (Figure 4). It should be noted that these predicted binding poses and interaction fingerprints are derived from computational docking and await experimental confirmation. This computational pipeline efficiently narrowed the chemical space to a set of candidates that are predicted to engage the Y89-W99 pocket with favorable geometry and binding energetics, providing a focused collection for experimental validation (Table 1).

**Table 1:** Predicted binding affinity and interaction profile of small molecules with the Y89-W99 site.

Name	PubChem ID	Affinity kcal/mol	rDock Score	Hydrophobic Contacts	π–π Interactions	Hydrogen Bonds
OTUi-1	1088469	−9.2	−25.7	Val82, Arg92, Ser101, Thr102, Ile118, His146	His146	-
OTUi-2	8023393	−8.8	−22.1	Val82, Tyr89, Arg92, Thr102, His146	His146	-
OTUi-3	1045968	−8.4	−21.0	Trp99, Ser101	Trp99 (two contacts)	-
OTUi-4	713096	−6.7	−24.0	Val82, Tyr89, Ser101	Tyr89	-
OTUi-5	2974206	−6.1	−15.8	Trp99, Ser101	-	Ser101
OTUi-6	890662	−6.6	−27.0	Tyr89, Trp99	Tyr89	-
OTUi-7	778345	−5.7	−27.2	Val82, Tyr89, Arg92, Trp99,	Trp99 (four contacts)	Trp99
OTUi-8	804043	−5.5	−26.9	Val82, Ser101	-	Thr102
OTUi-9	774975	−6.8	−23.2	Val82, Tyr89, Arg92,	-	Arg92
OTUi-10	349435	−5.6	−14.2	Val82	-	Glu104
OTUi-11	2339359	−6.3	−22.6	Val82	-	Arg92
OTUi-12	272514	−6.7	−18.2	Val82, Tyr89, Arg92, Ser101,	Tyr89	Glu104
OTUi-13	1910302	−7.5	−32.9	Trp99, Ser101, His146, Phe152	His146	Thr102
OTUi-14	664517	−8.7	−23.2	Tyr89, Trp99, Ser101	Tyr89, Trp99 (two contacts),	-
OTUi-15	4653365	−7.1	−24.9	Tyr89, Trp99	-	Trp99
OTUi-16	2545001	−6.5	−22.0	Ser101, His146	His146 (two contacts)	Arg92
OTUi-17	2407446	−6.1	−26.1	Val82, Ser101	-	Ser101
OTUi-18	664624	−7.0	−17.6	Ser101	-	Arg92, Ser101
OTUi-19	6871359	−7.6	−19.2	Val82, Tyr89, Arg92, Trp99, Ser101,	Trp99 (two contacts)	Glu78
OTUi-20	6356140	−7.5	−22.5	Trp99, Ser101	Trp99 (two contacts)	Arg92
OTUi-21	1397082	−7.4	−22.5	Val82, Trp99	Trp99 (three contacts)	-
OTUi-22	1084720	−6.2	−19.8	Val82, Trp99, Ser101	Trp99	Glu78 (two bonds)

AutoDock Vina binding affinities and rDock scores for the 24-compound library are summarized in Table 1. Vina affinities ranged from −5.5 kcal/mol (OTUi-8) to −9.2 kcal/mol (OTUi-1), with a median of −6.7 kcal/mol. The rDock scores spanned −14.28 (OTUi-10) to −32.98 (OTUi-13). The two scoring functions captured different features of the binding event, where Vina estimated total free energy of binding using an empirical scoring function, while rDock relied on a force-field-based sum of intermolecular terms that emphasized shape and electrostatic complementarity. Consistent with the known modest correlation between these metrics, no linear relationship was found between Vina affinity and rDock score. Consequently, Vina affinity was used as the primary docking metric for structure-activity analysis, while rDock scores provided complementary insight into conformational fit within the Y89-W99 cavity.

Ten of the 24 small molecules studied did not engage in π–π stacking; these included the two controls and several inhibitors with weak Vina affinities, such as OTUi-8 (−5.5 kcal/mol) and OTUi-10 (−5.6 kcal/mol). The remaining 14 compounds formed at least one π–π interaction, predominantly with Trp99, Tyr89, or His146, reflecting the highly aromatic nature of the pocket. The most frequently contacted residues across all interaction types were Ser101 (16/24 compounds), Val82 (15/24), Trp99 (12/24), Arg92 (11/24), and Tyr89 (10/24). Hydrophobic contacts dominated the intermolecular fingerprints, with hydrogen bonds playing a supportive role (Figure 4, Table 1).

### 2.4. Relationship Between Interaction Patterns and Affinity

Compounds with strong Vina affinities (≤−8.0 kcal/mol) were predicted to engage the pocket through π–π stacking with Trp99, Tyr89, or His146, together with extensive hydrophobic packing. However, as discussed below, exceptions such as OTUi-10 demonstrate that other binding modes can also produce potent inhibition. OTUi-1 (−9.2 kcal/mol) used a single His146 π-stack and a broad hydrophobic network involving His146, Ile118, Thr102, Ser101, Arg92, and Val82. OTUi-14 (−8.7 kcal/mol) formed three distinct π-stacks (two with Trp99, one with Tyr89) and hydrophobic contacts with Ser101, Trp99, and Tyr89. In contrast, weak binders such as OTUi-8 (−5.5 kcal/mol) and OTUi-10 (−5.6 kcal/mol) possessed only a single hydrophobic partner and one hydrogen bond, with no aromatic stacking. Hydrogen bonds alone were not sufficient to drive high affinity; the three strongest binders (affinities ≤ −8.4 kcal/mol) either lacked hydrogen bonds or used them sparingly, relying instead on shape complementarity and aromatic stacking. The rDock ranking highlighted optimal spatial fit. The top rDock score (−32.98, OTUi-13) resulted from a His146 π-stack, a Thr102 hydrogen bond, and a deeply buried hydrophobic anchor (Phe152, His146, Ser101, Trp99), yielding a Vina affinity of −7.5 kcal/mol. This demonstrates that a conformational complementarity can coexist with a moderate global free energy estimate.

### 2.5. Biochemical Validation of OTU Inhibitors in a Fluorometric DUB Assay

**Figure 5:**
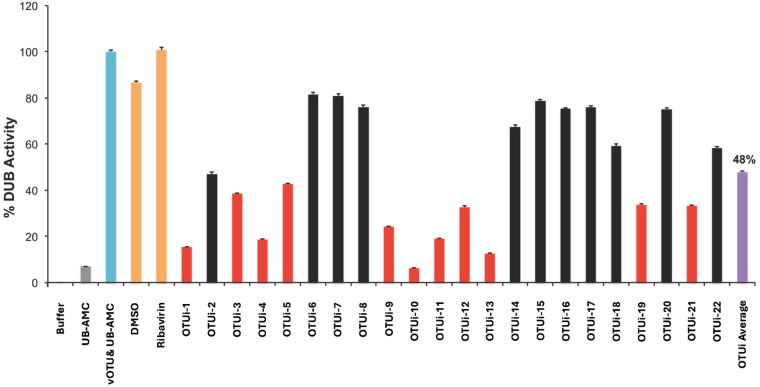
In vitro validation of novel CCHFV OTU inhibitors by fluorometric DUB assay. Inhibition of CCHFV OTU protease by putative small molecules from the in silico analysis, measured using the Ub-AMC cleavage assay. Initial velocities were converted to percent inhibition relative to vehicle (DMSO) control. *n* = 3.

The inhibitory activity of selected in silico hits was assessed using a quantitative Ub-AMC cleavage assay. Recombinant viral OTU protease was incubated with 0.1 μM Ub-AMC substrate in the presence of 20 μM concentrations of each test compound, and the release of fluorescent AMC was monitored (Figure 5). Ribavirin and DMSO were used as negative controls. Several small molecules exhibited strong inhibition, reflected by markedly reduced residual DUB activity. The most potent inhibitors included OTUi-10 (~7% residual activity; ~93% inhibition), OTUi-13 (~87% inhibition), and OTUi-1 (~85% inhibition). Additional high-performing hits were OTUi-4 and OTUi-11 (~81% inhibition), and OTUi-9 (~76% inhibition). A second tier of moderate inhibitors included OTUi-12 (~67% inhibition), OTUi-19 and OTUi-21 (each ~66% inhibition), and OTUi-5 (~57% inhibition). In contrast, several small molecules retained high DUB activity (>70%), indicating weaker inhibitory effects. Overall, while all tested small molecules showed some degree of OTU inhibition, a subset achieved >75–90% suppression at 20 µM, with an average inhibition of ~48% across the library (Figure 5). It is important to note that the percentage inhibition values represent single-point measurements at 20 μM compound concentration; full dose–response curves and IC50 determinations were not performed within the scope of this screening study. These experiments suggest that the Y89-W99 pocket may be a pharmacologically tractable site and demonstrate that the identified small molecules can effectively block OTU enzymatic function in vitro, laying the groundwork for further optimization and cellular testing. It is important to note that these inhibition measurements were obtained using purified recombinant OTU in a cell-free system. Thus, whether the compounds exhibit similar activity in a cellular context remains to be determined.

The rank order of biochemical inhibition did not mirror either the Vina or rDock ranking. The most striking example was OTUi-10, which achieved the highest inhibition despite a poor Vina affinity (−5.6 kcal/mol) and the lowest rDock score (−14.28). Nonetheless, all compounds achieving ≥75% inhibition harbored at least one π–π interaction with Trp99, Tyr89, or His146 (or, in the case of OTUi-9, a strategic hydrogen bond with Arg92), underscoring the essential role of specific polar and aromatic engagement in driving potent OTU blockade.

### 2.6. Structure and Activity Analysis

To integrate the affinity, rDock, and in vitro data, the small molecules were examined by chemotype. Unsubstituted phthalimide OTUi-10 delivered the strongest inhibition (93%) despite a weak Vina affinity (−5.6 kcal/mol) and the poorest rDock score (−14.28). Its interaction set was minimal (Glu104 H-bond, Val82 hydrophobic contact), suggesting a compact binding mode that was highly efficient within the sub-pocket but not accurately captured by in silico scoring. N-Phenethyl substitution (OTUi-4) introduced a Tyr89 π-stack and hydrophobic contacts with Ser101, Tyr89, and Val82, raising the Vina affinity to −6.7 kcal/mol (rDock −24.04) while retaining 81% inhibition. An ethoxymethylene-benzyl extension (OTUi-11) provided an Arg92 H-bond, improving Vina affinity to −6.3 kcal/mol (rDock −22.67) and also delivering 81% inhibition. N-Benzylphthalimide (OTUi-12) showed a Tyr89 π-stack and a broader hydrophobic profile (Ser101, Arg92, Tyr89, Val82) plus a Glu104 H-bond, resulting in a Vina affinity of −6.7 kcal/mol but a lower rDock score (−18.21) and moderate inhibition (67%). Thus, N-alkyl modifications that introduce aromatic or hydrogen-bond contacts tended to improve both scoring metrics and cellular potency, although OTUi-10 stands out as a distinct minimal pharmacophore.

Thiourea/benzothiadiazole series included OTUi-7, OTUi-8, OTUi-16 and OTUi-17. Small molecules without extended aromatic systems (OTUi-7, OTUi-8) fail to form productive π-stacks. OTUi-7 made four π–π contacts with Trp99 yet had a weak Vina affinity (−5.7 kcal/mol) and moderate rDock score (−27.29), indicating that unfavorable geometry or desolvation penalties can offset multiple aromatic contacts. OTUi-8 lacked π-stacking entirely and showed a similarly weak affinity (−5.5 kcal/mol; rDock −26.97). Acetylphenyl-quinoline thiourea (OTUi-16) engaged His146 via two π-stacks and an Arg92 H-bond, improving Vina affinity to −6.5 kcal/mol (rDock −22.09). Removal of the acetyl group (OTUi-17) abolished π-stacking and reduced affinity (−6.1 kcal/mol; rDock −26.13), confirming that large, planar aryl tails were required for productive His146 engagement.

Naphthoquinone–benzotriazole hybrids included OTUi-3 and OTUi-21. The meta-benzoic acid isomer OTUi-3 (−8.4 kcal/mol; rDock −21.02) formed two π–π stacks with Trp99 and hydrophobic contacts with Ser101 and Trp99. Its para-counterpart OTUi-21 (−7.4 kcal/mol; rDock −22.55) generated three π–π stacks with Trp99 and contacted Trp99 and Val82. Despite the higher number of π-interactions in OTUi-21, the meta arrangement yielded a ~1 kcal/mol more favorable Vina score, suggesting that subtle differences in carboxylate orientation fine-tuned electrostatic complementarity and/or solvation beyond what the interaction fingerprint alone captures. Both isomers showed moderate inhibition (~66%) but were not among the top hits.

Large polycyclic aromatics included OTUi-1, OTUi-2, OTUi-5, OTUi-9, OTUi-13, OTUi-14 and OTUi-15. Extended π-systems consistently yield high Vina affinities (≥−7.5 kcal/mol) by engaging His146, Trp99, or Tyr89. OTUi-1 (−9.2 kcal/mol; rDock −25.73, 85% inhibition) used a His146 π-stack and an extensive hydrophobic envelope. OTUi-2 (−8.8 kcal/mol; rDock −22.12) and OTUi-5 (−8.4 kcal/mol; rDock −21.02; 57% inhibition) relied on multiple Trp99 π-stacks. OTUi-13 (−7.5 kcal/mol; rDock −32.98) was noteworthy. Despite a moderate Vina affinity, it achieved the best rDock score and 87% inhibition, demonstrating that optimal spatial complementarity can translate into strong biochemical activity. OTUi-9 (−6.8 kcal/mol; rDock −23.23; 76% inhibition) lacked π-stacking altogether and instead relied on an Arg92 H-bond and hydrophobic contacts (Arg92, Tyr89, Val82). Its potent inhibition, comparable to that of compounds with multiple π-stacks, indicated that a strategically placed hydrogen bond and a hydrophobic ensemble can compensate for the absence of aromatic stacking in this pocket.

In short, π-stacking with Trp99, Tyr89, or His146 was a common feature among many compounds with high Vina affinity and strong inhibition, suggesting it may contribute favorably to binding. However, this trend was not absolute, as discussed below for OTUi-10. Hydrophobic contacts amplified binding (≥5 contacts typically add 1–2 kcal/mol), while hydrogen bonds fine-tuned interactions and, in the case of OTUi-9, rescued potent inhibition in the absence of stacking. The rDock scoring function, although not superior for ranking affinities, effectively identifies molecules with optimal conformational fit (e.g., OTUi-13), some of which exhibited excellent biochemical activity. The prominent outlier OTUi-10, a minimal phthalimide, highlights that very small, polar scaffolds can achieve near-complete target engagement despite unfavorable in silico scores. Thus, while π–π stacking appears to be a favorable interaction for many of the tested compounds, it is not a strict requirement for potent OTU inhibition. The binding mode of OTUi-10, which lacks such interactions, warrants further investigation through co-crystallization or other biophysical methods. These findings validate the Y89-W99 pocket as a druggable site and nominate OTUi-10, OTUi-13, and OTUi-1 as priority hit compounds for further optimization with distinct structural vectors for further optimization.

### 2.7. In Silico Drug-Likeness and Toxicity Profiling

The 22 OTUi compounds were evaluated for key physicochemical properties and toxicity risks (Table 2). Applying the stringent filters for drug-likeness (MW < 450 Da, cLogP < 5, cLogS > −4, PSA < 60 Å^2^, and drug-likeness score > 0) revealed a substantial attrition of the library. All compounds satisfied the molecular weight (<450 Da) and lipophilicity (<5) criteria. However, only nine compounds (OTUi-4, OTUi-6, OTUi-7, OTUi-8, OTUi-10, OTUi-11, OTUi-12, OTUi-18, OTUi-20) displayed cLogS > −4, indicative of acceptable predicted solubility. Polar surface area proved the most restrictive parameter: only eight molecules (OTUi-2, OTUi-4, OTUi-6, OTUi-9, OTUi-10, OTUi-11, OTUi-12, OTUi-15) fell below 60 Å^2^, a threshold associated with good membrane permeability. Sixteen compounds returned a positive drug-likeness score (>0), and the remaining six (OTUi-2, OTUi-3, OTUi-5, OTUi-13, OTUi-15, OTUi-21) contained fragment-based alerts common to non-drug-like structures. Stringent application of all five criteria simultaneously reduced the library to only four fully compliant molecules: OTUi-4, OTUi-10, OTUi-11, and OTUi-12.

Notably, the most potent biochemical inhibitor, OTUi-10, passes every drug-likeness filter, reinforcing its attractiveness as a minimal, developable scaffold. The second- and third-most potent inhibitors, OTUi-13 and OTUi-1, each failed at least two criteria: OTUi-13 has poor predicted solubility (cLogS = −5.7), a PSA of 83.6 Å^2^, and a negative drug-likeness score (−2.6), while OTUi-1 (cLogS −4.8, PSA 79.6 Å^2^) fails the solubility and PSA cutoffs. This discordance between target-based potency and computed drug-likeness underscores the need to balance early hit activity with developability properties. Thus, both OTUi-1 and OTUi-13 may require prodrug strategies or formulation approaches to mitigate solubility limitations. Other active inhibitors, such as OTUi-14 (MW 424.5 Da, cLogP 2.6, but PSA 70.8 Å^2^ and cLogS −5.3) and OTUi-19 (PSA 137.6 Å^2^, cLogS −5.4, mutagenic), likewise exhibit at least one drug-likeness deficiency.

Toxicity predictions further narrowed the candidate pool. Nine compounds showed no mutagenic, tumorigenic, or reproductive toxicity alerts: OTUi-2, OTUi-4, OTUi-5, OTUi-6, OTUi-10, OTUi-11, OTUi-12, OTUi-14, and OTUi-18. Importantly, the four molecules that met all drug-likeness filters OTUi-4, OTUi-10, OTUi-11, and OTUi-12 were all free of toxicity flags, providing a set of clean, rule-compliant starting points. The high-potency inhibitor OTUi-9 (76% inhibition) carried a reproductive toxicity alert and failed solubility (cLogS −5.0). OTUi-13, despite its strong inhibition, was flagged as mutagenic in addition to its physicochemical shortcomings. These data advocated prioritizing OTUi-10, OTUi-11, and OTUi-4 for initial hit-to-lead optimization efforts, given their combined potency and favorable in silico ADMET profiles, while treating OTUi-1 and OTUi-13 as high-activity templates that may require property-tailored chemical modifications.

## 3. Discussion

The CCHFV OTU protease is widely recognized as a linchpin of innate immune evasion and a high-value antiviral target, yet the development of drug-like inhibitors has been repeatedly stymied by a shallow active-site architecture that engenders poor selectivity and by a persistent gap between biochemical potency and cellular efficacy. In this study, we integrate a robust recombinant OTU production pipeline with structure-guided virtual screening against the previously identified Y89-W99 exosite [22] and identify a novel cluster of small molecules that inhibit the deubiquitinase activity of the OTU, several of which achieve >85% suppression at a concentration of 20 µM. The work provides a conceptual and experimental framework that shifts the therapeutic targeting strategy for CCHFV OTU from the canonical catalytic cleft to a functionally critical remote pocket, offering a fresh solution to the selectivity conundrum that has plagued earlier inhibitors. The conclusion that these compounds target the Y89-W99 pocket is currently based on molecular docking predictions and indirect functional inhibition; direct experimental evidence of binding is not yet available.

The most salient mechanistic insight to emerge from our data is the demonstration that small molecules predicted to occupy the Y89-W99 pocket can profoundly suppress OTU catalytic activity in vitro, despite this pocket lying distal to the D37-C40-H151 catalytic triad. A plausible explanation, consistent with the structural biology of the OTU-ubiquitin complex [12], is that the Y89-W99 region forms an integral part of the extended ubiquitin/ISG15 docking interface, and that ligand binding at this site sterically occludes substrate recognition or allosterically perturbs the conformations of active-site loops required for hydrolysis. This mode of inhibition is fundamentally different from that of active-site-directed inhibitors such as the 2-aminothiazole series [18], which engage the catalytic cysteine and inevitably compete with the highly conserved C-terminal LRLRGG motif of ubiquitin and ISG15, thereby incurring cross-reactivity with host deubiquitinases that recognize the same motif. The existence of a functional exosite on the OTU has precedence in the behavior of the engineered ubiquitin variant CC4, which binds an overlapping surface and blocks viral RNA synthesis through a mechanism that is not fully explained by simple occlusion of the active site [19]. Our data now suggest that the Y89-W99 pocket may be chemically tractable with drug-like small molecules, based on computational predictions and biochemical inhibition. However, direct confirmation of pocket engagement and the resulting functional effects requires further experimental validation. Detailed kinetic experiments will be required to determine whether the inhibition is purely competitive with respect to the ubiquitin/ISG15 substrate, non-competitive, or uncompetitive, but the exosite location strongly favors a non-active-site-competitive mechanism, a property that could be exploited to develop inhibitors that are effective even at high local substrate concentrations encountered during infection.

Early discovery campaigns yielded inhibitors such as homidium bromide and phenanthrenequinone that were identified through docking and exhibited dose-dependent activity in Ub-AMC assays [17]. A subsequent medicinal chemistry effort produced a competitive inhibitor with an IC_50_ of 10.7 µM, but its broad-spectrum inhibition of human DUBs (USP7, UCHL5, OTUD1, OTUD7B) underscored the intrinsic difficulty of achieving selectivity when targeting the ubiquitin-binding groove [18]. Our study departs from this trajectory in a critical way: rather than iterating on the active-site pharmacophore, we leverage a structurally distinct pocket that we previously showed to be under strong conserved constraint in nairoviruses yet divergent in eukaryotic OTU-family proteases [22]. The consequence is a set of inhibitor scaffolds that are, by design, anchored to a viral-specific surface patch. While explicit counter-screens against human DUBs remain to be performed, the phylogenetic and structural rationale provides a testable hypothesis for selective inhibition that none of the earlier small-molecule chemotypes could offer. Moreover, unlike protein-based antagonists such as CC4, whose intracellular delivery requires adenoviral vectors and which failed to confer survival benefit in a lethal mouse model [20], small molecules with favorable predicted drug-likeness (Table 2) present a far more straightforward path toward pharmacological optimization and in vivo dosing. In this respect, the present scaffolds represent a clear advancement in terms of potential translational tractability.

We show that ectopic expression of the CCHFV OTU in HEK293T cells globally reduces the abundance of both poly- and mono-ubiquitinated proteins, directly corroborating earlier studies that mapped the OTU’s immunomodulatory function to its deubiquitinase activity [14,15]. This result is significant in two respects: first, it confirms that the refolded recombinant enzyme used for inhibitor screening faithfully recapitulates the catalytic properties of the native viral protein inside mammalian cells; second, it establishes a convenient cellular model in which the ability of candidate inhibitors to restore ubiquitin-dependent signaling can be evaluated in a BSL-2 setting. The critical next step, therefore, is to test whether the top-performing compounds from our biochemical screen can reverse OTU-mediated deubiquitination and, ideally, rescue NF-κB or interferon-β reporter activity. Such cellular target-engagement data would serve as a crucial bridge between the in vitro inhibition reported here and eventual antiviral efficacy testing in CCHFV infection models. The absence of such data at this stage is a deliberate reflection of the early-stage nature of the program, and we caution against extrapolating the single-concentration biochemical inhibition to in vivo protection.

Deconstruction of the protein–ligand contact fingerprints revealed that the Y89-W99 pocket is primarily an aromatic–hydrophobic groove. For the majority of compounds tested, π–π stacking with Trp99, Tyr89, or His146 was associated with higher Vina affinity and stronger biochemical inhibition. However, the most potent inhibitor in our screen, OTUi-10, achieved ~93% inhibition without forming any π–π interactions, demonstrating that alternative binding modes can also be highly effective. Therefore, while aromatic stacking appears to be a favorable feature for many chemotypes, it is not an absolute requirement for potent OTU blockade. All compounds achieving ≥75% inhibition engaged at least one of these residues via π-stacking or, in the unique case of OTUi-9, via a well-placed Arg92 hydrogen bond. Hydrophobic contacts with Ser101, Val82, and Arg92 further amplified binding, with inhibitors harboring five or more such contacts gaining 1–2 kcal mol^−1^ in predicted affinity. Notably, multiple π-contacts alone were insufficient. OTUi-7 formed four Trp99 stacks yet bound weakly (−5.7 kcal mol^−1^), indicating that stacking geometry and desolvation costs can offset the benefits of multiple aromatic interactions. Structure dissection by chemotype reinforced these trends: in the phthalimide series, the minimal OTUi-10 scaffold achieved remarkable inhibition despite weak in silico scores, while N-alkyl extensions that introduced aryl or hydrogen-bonding moieties improved docking metrics without compromising potency. The thiourea series demonstrated that large, planar aryl tails are required to reach His146, and the naphthoquinone–benzotriazole pair highlighted the sensitivity of carboxylate orientation. Together, the data define a pharmacophore in which an aromatic anchor (preferably engaging Trp99 or His146), surrounded by a complementary hydrophobic collar, is the dominant driver of OTU inhibition. The outlier performance of OTUi-10 further suggests that a compact, polar scaffold may access a deep sub-pocket not yet fully exploited by the larger polycyclic compounds. Collectively, these preliminary SAR observations are hypothesis-generating and require confirmation through dose–response studies, biophysical binding assays, and structural biology.

The ADMET profiling reinforced that early drug-likeness and toxicity filtering was essential for hit triage, as only four of 22 compounds satisfied all five criteria. Reassuringly, the clean set included OTUi-10, our most potent inhibitor in the single-concentration screen, as well as OTUi-11 and OTUi-4, suggesting that favorable predicted drug-likeness can coincide with promising biochemical inhibition. However, experimental validation of solubility, permeability, and metabolic stability remains necessary to confirm these in silico predictions. The phthalimide scaffold of OTUi-10, with its minimal size and favorable solubility, offers an attractive starting point for medicinal chemistry expansion. In contrast, high-activity but property-deficient hits such as OTUi-1 and OTUi-13 could be progressed through rational modification to improve solubility and eliminate structural alerts. Follow-up in vitro genotoxicity and ADME assays will be necessary to confirm these predictions and guide the selection of a safe, orally bioavailable lead candidate.

The identification of potent inhibitors targeting the Y89-W99 pocket of CCHFV OTU is significantly bolstered by the discovery that malarial parasites (*P. falciparum*, *P. vivax*, and *P. yoelii*) possess previously uncharacterized viral OTU-like proteins with nearly identical inhibition pockets [23,24]. Structural modeling and multiple sequence alignment reveal that these malarial OTU proteins (mOTUs) harbor the same highly conserved Y-WG residues that define the CCHFV OTU inhibition pocket. The efficacy of the same small-molecule library across both viral and parasitic proteases underscores the pharmacological tractability of this specific exosite. For instance, CID 349435 (identified as OTUi-10 in this study and #65 in the malarial study) emerged as a premier lead in both contexts, achieving over 93% inhibition of CCHFV OTU and an IC50 of 0.3 µM against pfOTU and 0.03 µM against pvOTU [23]. Similarly, CID 2339359 (OTUi-11/#66) showed robust activity against both CCHFV and multiple Plasmodium species. This cross-species effectiveness suggests that inhibitors targeting the Y89-W99 aromatic–hydrophobic groove are not merely narrow-spectrum antivirals, but may represent a new class of broad-spectrum protease inhibitors. While CCHFV and Plasmodium are biologically distinct, they utilize these conserved OTU domains to antagonize host innate immunity by deubiquitinating key signaling molecules like NF-κB. The ability of these scaffolds to block DUB activity in both a high-fatality virus and a global parasite pathogen confirms the functional criticality of the Y-W pocket. Future development of these leads, particularly the rule-compliant OTUi-10 and OTUi-11, should leverage this structural conservation to design therapies capable of addressing multiple intracellular pathogens that exploit the OTU deubiquitinase system.

An unanticipated finding was the wide range of inhibitory activities within a structurally clustered set of compounds that were all predicted to occupy the same Y89-W99 pocket. While some molecules, such as OTUi-10 and OTUi-13, nearly abolished OTU activity, others with similar docking scores showed only modest effects. This dispersion could be interpreted in several ways: it may reflect differential solubility or aggregation of the compounds under assay conditions, differences in cell permeability (for the few compounds that may enter cells), or genuine but subtle structural determinants of binding that are not captured by the static docking algorithm. From a drug-discovery perspective, this observation underscores the necessity of multipoint dose–response profiling and orthogonal biophysical assays before inferring structure-activity relationships. It also reinforces the notion that the Y89-W99 pocket, while clearly capable of accommodating inhibitory chemotypes, likely possesses nuanced topological and dynamic features that will only become apparent through co-crystal structures of the OTU with inhibitors.

Several recent studies have advanced the field of CCHFV OTU inhibitor discovery, providing important context for our findings. Beckmann et al. [18] identified and optimized a 2-aminothiazole series, achieving a competitive inhibitor with an IC_50_ of 10.7 µM in a Ub-rhodamine-110 assay; however, this compound also inhibited several human DUBs, revealing a selectivity liability that underscores the challenge of targeting the active site directly. This aligns with the rationale for our exosite-directed approach, which targets the non-catalytic Y89-W99 pocket to potentially circumvent such off-target effects. In parallel, computational drug-repurposing campaigns have nominated promising candidates: Hashim et al. [21] identified paromomycin as a top hit against the OTU protease through in silico screening of FDA-approved drugs, while the same group identified 1,3,6-trigalloyl glucose as a high-affinity natural product inhibitor (XP GScore: −9.351 kcal/mol) forming strong interactions with key catalytic residues [25]. Additionally, Hassam et al. [26] employed computational and molecular dynamics-driven discovery to identify *Moringa oleifera* peptides targeting the RdRp-embedded OTU protease. These complementary approaches ranging from synthetic medicinal chemistry to natural product and peptide discovery highlight the growing interest in OTU-targeted antivirals and reinforce the value of our structure-guided screening strategy.

Beyond computational and biochemical studies, recent in vivo work has addressed the critical translational gap in CCHFV OTU inhibitor development. Scholte et al. [20] evaluated adenovirus-mediated delivery of the ubiquitin variant CC4, a potent and selective protein-based OTU inhibitor, in a lethal CCHFV mouse model. Although Ad-CC4 delivery was feasible, it failed to protect mice from lethal CCHFV challenge, highlighting the substantial hurdles in translating protein-based inhibitors to in vivo efficacy and reinforcing the need for small-molecule alternatives. Encouragingly, Liu et al. [27] demonstrated that baloxavir sodium, an influenza antiviral, exhibits potent anti-CCHFV activity in vitro and significantly improves survival rates while reducing tissue viral loads in vivo. Although baloxavir targets the viral polymerase rather than the OTU protease, this study provides a proof-of-concept that small-molecule inhibitors can achieve efficacy in CCHFV animal models. Collectively, these recent findings underscore both the promise and the challenges of developing OTU-targeted antivirals, and they provide a clear roadmap for the next phase of our work: advancing the lead compounds identified here, particularly OTUi-10, OTUi-4, and OTUi-11, through dose–response profiling, selectivity screening, and ultimately, in vivo efficacy studies.

We wish to articulate the limitations of this study with precision, as they directly inform the most productive next steps. First, all compound activities are based on a single inhibitor concentration (20 μM); IC50 values, Hill coefficients, and kinetic parameters are not available. Consequently, potency comparisons with literature compounds, including the previously reported 2-aminothiazole inhibitor (IC50 = 10.7 μM), are not justified. Second, no experimental evidence, such as site-directed mutagenesis of Y89 or W99, chemical shift perturbation by NMR, or direct binding measurements, confirms that the inhibitors physically engage the Y89-W99 pocket, and alternative binding modes cannot be excluded. Third, selectivity over human DUBs remains entirely untested; the phylogenetic argument, while logical, is not a substitute for experimental data. Fourth, inhibition of the OTU’s deISGylase activity was not examined, leaving open the question of whether these compounds can antagonize both arms of the OTU’s innate immune evasion function. Fifth, the recombinant OTU was purified under denaturing conditions and refolded; although the high solubility (>70%) and robust cellular activity argue against large-scale misfolding, subtle structural anomalies could influence inhibitor binding. Sixth, while the cellular ubiquitination assay confirms that the recombinant OTU protease is functionally active in a mammalian context, the inhibitors themselves were not tested in cells. Whether the lead compounds can restore global ubiquitination levels or rescue downstream innate immune signaling remains unknown and represents a critical limitation of the present study. Seventh, while the cellular ubiquitination assay demonstrates that the recombinant OTU protease is functionally active in a mammalian context, we have not yet tested whether the identified inhibitors can reverse OTU-mediated deubiquitination or restore innate immune signaling in cells. Such cellular target engagement studies represent a critical bridge between the biochemical inhibition reported here and eventual antiviral efficacy testing. Eighth, direct biophysical evidence of compound binding such as surface plasmon resonance, isothermal titration calorimetry, differential scanning fluorimetry, or co-crystallization is absent from this study. The proposed binding mode within the Y89-W99 pocket therefore remains computationally predicted and indirectly supported by enzymatic inhibition data, rather than experimentally confirmed. Finally, all drug-likeness and toxicity assessments were performed in silico and must be validated experimentally.

The data presented here chart a clear, hierarchical research agenda. In the immediate term, full dose–response curves and kinetic analyses should be generated for the top five inhibitors, alongside counter-screens against representative human DUBs (USP7, UCHL5, OTUD1, OTUD7B) to test the selectivity hypothesis. Biophysical validation of direct binding should be pursued through surface plasmon resonance, isothermal titration calorimetry, or microscale thermophoresis. Mutational analysis in which the Y89-W99 pocket is disrupted (e.g., Y89A, W99A) and the effect on inhibitor potency is quantified will be essential to confirm the binding site. Co-crystallization trials with OTUi-10 or OTUi-13 should be initiated to provide the first high-resolution structure of a drug-like ligand bound to the CCHFV OTU, enabling true structure-guided optimization.

Simultaneously, the cellular OTU-expression system established here should be employed to assess the permeability and intracellular target engagement of the most promising compounds, using restoration of high-molecular-weight ubiquitin conjugates as a primary readout. Inhibitors that demonstrate cellular activity should then be profiled in deISGylase cleavage assays (ISG15-AMC and cellular ISGylation readouts) to verify dual-activity blockade. Compounds meeting these criteria will be advanced to ADME-Tox profiling, including metabolic stability in liver microsomes, plasma protein binding, and cytotoxicity in primary human cell lines. Only upon completion of these milestones will it be appropriate to evaluate antiviral efficacy in CCHFV infection models under appropriate biosafety containment.

## 4. Materials and Methods

### 4.1. Recombinant Expression and Purification of CCHFV OTU

The coding sequence of the CCHFV OTU protease domain was optimized for *Escherichia coli* codon usage and synthesized with a C-terminal hexahistidine tag (GenBank DQ211623.1) as described previously [17]. The synthetic gene was inserted into the pET-26b(+) vector (Novagen, Darmstadt, Germany) using NcoI and NdeI restriction sites (New England Biolabs, Ipswich, MA, USA). The resulting plasmid was transformed into BL21(DE3) competent cells (New England Biolabs, Ipswich, MA, USA). For protein production, an overnight starter culture was diluted 1:100 in fresh LB medium (Merck, Darmstadt, Germany) containing kanamycin (50 µg mL^−1^) (Sigma-Aldrich, St. Louis, MO, USA) and grown at 37 °C with shaking until the optical density at 600 nm reached 0.6. Expression was induced by adding isopropyl β-D-1-thiogalactopyranoside (IPTG) (Thermo Fisher Scientific, Waltham, MA, USA) to a final concentration of 1 mM, and incubation was continued for 16 h at 25 °C. Cells were harvested by centrifugation at 5000× *g* for 15 min at 4 °C, and the pellet was stored at −80 °C. Frozen cell pellets were resuspended in solubilization buffer (8 M urea, 20 mM Tris-HCl pH 8.0) and lysed by sonication on ice (5 min total, 10 s pulses with 20 s pauses). The lysate was clarified by centrifugation at 20,000× *g* for 30 min at 4 °C and filtered through a 0.45 µm membrane. The His-tagged OTU protein was purified by immobilized metal-ion affinity chromatography using a HisTrap HP 1 mL column connected to an ÄKTAprime plus system (GE Healthcare, Uppsala, Sweden). Equilibration was performed with a binding buffer (8 M urea, 500 mM NaCl, 50 mM sodium phosphate, 20 mM imidazole, 14.1 µM β-mercaptoethanol, pH 8.0). After sample loading and washing, bound protein was eluted with a linear gradient of elution buffer (binding buffer containing 500 mM imidazole). The peak fraction containing OTU was identified by UV absorbance at 280 nm and analyzed by SDS-PAGE (12% gel, Coomassie brilliant blue R-250 staining, Bio-Rad Laboratories, Hercules, CA, USA.

### 4.2. Western Blot Confirmation

After SDS-PAGE, proteins were electrotransferred to a nitrocellulose membrane (Bio-Rad Laboratories, Hercules, CA, USA) (350 mA for 1 h at 4 °C). The membrane was blocked with 5% non-fat dry milk in Tris-buffered saline containing 0.1% Tween-20 (TBST) and then incubated overnight at 4 °C with an anti-His tag monoclonal antibody (1:1000 dilution) as described previously [23]. Following three washes with TBST, the membrane was incubated with horseradish peroxidase-conjugated secondary antibody (1:2000) for 1 h at room temperature. Signals were developed with enhanced chemiluminescence substrate (Bio-Rad Laboratories, Hercules, CA, USA) and recorded with a ChemiDoc MP imaging system (Bio-Rad Laboratories, Hercules, CA, USA).

### 4.3. Refolding and Solubility Assessment

The imidazole-containing eluate (containing purified OTU in denaturing buffer with 8 M urea) was first subjected to buffer exchange into phosphate-buffered saline (PBS) using a desalting column (10 kDa molecular-weight cutoff) (Sartorius AG, Göttingen, Germany). Then, 25 mL of the resulting protein solution was diluted into 475 mL of PBS (final volume 500 mL) to reduce the denaturant concentration and promote protein refolding. The final urea concentration after dilution was approximately 0.4 M. The diluted sample was incubated at 4 °C for 1 h to allow refolding. To evaluate solubility, a sample of the concentrated protein was centrifuged at 14,000× *g* for 5 min, and both the supernatant and pellet were analyzed by SDS-PAGE and Coomassie staining. Band intensities were quantified to estimate the percentage of soluble protein.

### 4.4. Construction of Mammalian Expression Vectors and Cell Transfection

To examine OTU activity in a cellular context, the OTU coding region was excised from pET-26b(+) using XbaI and EcoRI and ligated into the pcDNA3.1(+) vector (Invitrogen, Waltham, MA, USA) that had been linearized with NheI and EcoRI and dephosphorylated. HEK293 cells were maintained in DMEM supplemented with 10% fetal bovine serum and 1% penicillin/streptomycin. For transfection, 4 × 10^4^ cells per well were seeded in a 6-well plate one day before the experiment. Polyethyleneimine (PEI) (Polysciences, Warrington, PA, USA) was used as the transfection reagent: 2 µg of plasmid DNA and 2 µg of PEI were mixed in 200 µL of serum-free medium, incubated for 15 min at room temperature, and added dropwise to the cells. Cells were harvested 48 h post-transfection for downstream analysis.

### 4.5. Analysis of Cellular Ubiquitin Conjugates by Western Blot

Transfected HEK293 cells (ATCC CRL-1573, ATCC, Manassas, VA, USA) were lysed in a RIPA buffer containing 1 mM phenylmethylsulfonyl fluoride. Protein concentrations were determined, and equal amounts of total protein were resolved by SDS-PAGE and transferred to nitrocellulose membranes. Ubiquitinated proteins were detected with two distinct primary antibodies: a polyubiquitin-specific antibody (BML-PW8805) and an antibody recognizing both mono- and polyubiquitin conjugates (BML-PW8810), each at a dilution of 1:1000 (Enzo Life Sciences, Farmingdale, NY, USA). Incubations with primary antibodies were carried out overnight at 4 °C, followed by HRP-conjugated secondary antibody and chemiluminescent detection. Band intensities for high-molecular-weight smears were quantified using ImageJ (ImageJ 1.54t), and the signals were normalized to β-actin.

### 4.6. In Silico Docking and Interaction Analysis

The crystal structure of the CCHFV OTU domain in complex with ubiquitin (PDB ID: 3PRP) was retrieved, and the ubiquitin molecule and crystallographic water molecules were removed. The protein was prepared in AutoDockTools (version 1.5.6) by adding polar hydrogen atoms and assigning Gasteiger charges. A docking grid of 22 × 20 × 20 Å was centered on the conserved Y89-W99 pocket as described previously [22]. A curated library of 22 small molecules with potential deubiquitinase-inhibitory activity based on the previous enrichment analysis [23] was docked into this grid using AutoDock Vina 1.1.2. In addition, The AceDock application accessible through the PlayMolecule web platform (https://playmolecule.org/AceDock/, accessed on 16 April 2026) was used to determine rDock score. rDock scoring function is a weighted sum of intermolecular terms (van der Waals, attractive and repulsive polar, aromatic, solvation, and rotatable bond penalties), ligand intramolecular terms, site intramolecular terms, and external restraint terms. Their binding poses were visualized to extract two-dimensional protein–ligand interaction diagrams using PoseEdit (https://proteins.plus, accessed on 16 April 2026). For each small molecule, the docking pose with the highest predicted binding affinity was selected and converted from PDBQT to SDF format using Open Babel (v3.1.1), with the addition of explicit hydrogen atoms [28]. The CCHFV OTU protein structure (PDB ID: 3PRP) and the ligand SDF files were uploaded to the ProteinsPlus platform. Two-dimensional interaction diagrams were generated using the JAMDA module, and the optimal representation for each complex was refined in PoseEdit [29,30,31]. Hydrogen bonds, hydrophobic contacts, π–π stacking interactions, and salt bridges were automatically detected on the basis of the geometric criteria built into PoseEdit. All interaction maps were exported in scalable vector graphics (SVG) format.

### 4.7. In Silico Drug-Likeness and Toxicity Assessment

The molecular properties of the tested OTU inhibitors were evaluated computationally using DataWarrior (version 6.1.0) software as we have done previously [32,33]. Parameters calculated included molecular weight, the octanol-water partition coefficient (clogP), aqueous solubility (clogS), topological polar surface area, and fragment-based drug-likeness scores. Toxicity risks were predicted for mutagenicity, tumorigenicity, irritant effects, and reproductive toxicity using the built-in risk assessment algorithms.

### 4.8. Fluorometric Deubiquitinase Activity Assay

The enzymatic activity of the recombinant OTU protease and its inhibition were monitored with a ubiquitin-7-amido-4-methylcoumarin (Ub-AMC) substrate (Boston Biochem, Cambridge, MA, USA). Assays were performed in a reaction buffer consisting of 10 mM HEPES, 100 mM NaCl, and 2.5 mM DTT (pH 7.5). The protease (6.25 nM final concentration) was pre-incubated with test small molecules (20 μM) or vehicle (DMSO) for 15 min at 25 °C. The reaction was initiated by adding Ub-AMC to a final concentration of 100 nM. Fluorescence intensity (excitation 360 nm, emission 460 nm) was recorded every 2 min over a period of 60 min using a microplate reader (Varioskan LUX, Thermo Fisher Scientific, Waltham, MA, USA) thermostatted at 25 °C. Initial velocities were determined from the linear portion of the progress curves and expressed as a percentage of the DMSO control. Due to the primary objective of identifying initial hit compounds rather than performing full pharmacological characterization, the study focused on single-concentration inhibition screening (20 μM) without determination of IC50 values or detailed kinetic parameters. These experiments are planned as part of future hit validation efforts.

### 4.9. Statistical Analysis

Quantitative data are presented as mean ± standard error of the mean (SEM) as indicated. Group differences were assessed by two-tailed Student’s *t*-test. A *p*-value < 0.05 was considered statistically significant. Densitometry was performed with ImageJ (version 1.54t).

## 5. Conclusions

This study provides a conceptual framework and preliminary biochemical data supporting further investigation of exosite-directed inhibition of the CCHFV OTU protease. By integrating computational targeting of the conserved Y89-W99 pocket with a sensitive biochemical assay, we have identified novel chemical matter that potently suppresses viral deubiquitinase activity, circumventing the selectivity liabilities inherent to active-site inhibition. The work illuminates a plausible allosteric mechanism that, if experimentally validated, could reshape the design philosophy for OTU-targeted antivirals. While the inhibitors described here are at an early discovery stage and await rigorous characterization, they offer a starting point for a medicinal chemistry campaign, though rigorous dose–response and selectivity profiling remain necessary before advancing toward clinical development against Crimean–Congo hemorrhagic fever. The stepwise validation pipeline outlined above provides a logical roadmap to translate these biochemical hits into agents worthy of testing in life-threatening infection. It must be emphasized that the inhibitors described here have not yet been evaluated in cellular or antiviral assays; therefore, their therapeutic potential remains hypothetical at this stage.

## Figures and Tables

**Table 2:** In silico drug-likeness and toxicity assessment of studied small molecules.

OTUi ID	Total MW	cLogP	cLogS	Polar Surface Area	Druglikeness	Mutagenic	Tumorigenic	Reproductive Effective
OTUi-1	401.5	2.5	−4.8	79.6	7.4	none	high	none
OTUi-2	383.4	4.9	−5.5	55.4	−1.7	none	none	none
OTUi-3	410.4	3.0	−6.0	114.2	−2.2	high	high	none
OTUi-4	265.3	2.8	−3.0	37.4	3.1	none	none	none
OTUi-5	432.3	3.5	−5.5	66.9	−2.6	none	none	none
OTUi-6	250.3	2.7	−3.4	45.2	−0.2	none	none	none
OTUi-7	292.4	3.7	0.3	101.4	−0.8	low	none	none
OTUi-8	330.4	4.1	−0.7	119.4	−0.1	high	high	none
OTUi-9	251.2	2.9	−5.0	52.3	1.9	none	none	high
OTUi-10	161.2	0.7	−2.0	46.2	1.9	none	none	none
OTUi-11	307.3	2.2	−3.2	46.6	2.2	none	none	none
OTUi-12	251.3	2.4	−2.9	37.4	2.8	none	none	none
OTUi-13	369.4	4.2	−5.7	83.6	−2.6	high	none	none
OTUi-14	424.5	2.6	−5.3	70.8	3.5	none	none	none
OTUi-15	355.2	3.2	−4.7	51.2	−2.3	low	none	none
OTUi-16	321.4	3.3	−5.2	86.1	−1.0	high	none	none
OTUi-17	313.8	4.0	−5.3	69.0	−1.1	none	none	low
OTUi-18	334.3	0.5	−3.5	79.6	4.5	none	none	none
OTUi-19	419.5	3.4	−5.4	137.6	7.2	high	none	none
OTUi-20	388.4	1.8	−3.9	66.9	3.3	none	none	high
OTUi-21	410.4	3.0	−6.0	114.2	−2.2	high	high	none
OTUi-22	346.4	2.4	−6.3	71.6	2.4	high	low	high

## Data Availability

All data generated or analyzed during this study are included in this published article.

## References

[1] Bente D.A., Forrester N.L., Watts D.M., McAuley A.J., Whitehouse C.A., Bray M. (2013). Crimean-Congo hemorrhagic fever: History, epidemiology, pathogenesis, clinical syndrome and genetic diversity. Antivir. Res..

[2] Şahan S., Topluoğlu S., Temel F., Coşgun Y., Öz E., Demirkol M.E., Birinci Ş. (2025). Evaluation of epidemiological characteristics of Crimean-Congo haemorrhagic fever patients reported to the National Surveillance System in Türkiye, 2011–2024. Acta Trop..

[3] Gale P., Stephenson B., Brouwer A., Martinez M., de la Torre A., Bosch J., Foley-Fisher M., Bonilauri P., Lindström A., Ulrich R. (2012). Impact of climate change on risk of incursion of Crimean-Congo haemorrhagic fever virus in livestock in Europe through migratory birds. J. Appl. Microbiol..

[4] Lindeborg M., Barboutis C., Ehrenborg C., Fransson T., Jaenson T.G., Lindgren P.-E., Lundkvist Å., Nyström F., Salaneck E., Waldenström J. (2012). Migratory birds, ticks, and Crimean-Congo hemorrhagic fever virus. Emerg. Infect. Dis..

[5] Yeşilbağ Z., Karadeniz A., Koçulu S., Kayhan C.B. (2020). Epidemiological characteristics, clinical and laboratory findings supporting preliminary diagnosis of Crimean-Congo hemorrhagic fever in an endemic region in Turkey. Wien. Klin. Wochenschr..

[6] Igan H., Hanci H. (2025). The six-year prevalence of Crimean-Congo hemorrhagic fever (CCHF) in Erzurum, Turkey. J. Vector Borne Dis..

[7] Dokuzoguz B., Celikbas A.K., Gok S.E., Baykam N., Eroglu M.N., Ergönül Ö. (2013). Severity scoring index for Crimean-Congo hemorrhagic fever and the impact of ribavirin and corticosteroids on fatality. Clin. Infect. Dis..

[8] Ertem G., Sönmezer M.Ç., Temocin F., Hatipoğlu Ç.A., Tülek N., Oral B. (2016). The efficacy of oral ribavirin on clinical and laboratory parameters in Crimean Congo hemorrhagic fever: An observational study from Turkey. Turk. J. Med. Sci..

[9] D’Addiego J., Elaldi N., Wand N., Osman K., Bagci B.K., Kennedy E., Pektas A.N., Hart E., Slack G., Hewson R. (2023). Investigating the effect of ribavirin treatment on genetic mutations in Crimean--Congo haemorrhagic fever virus (CCHFV) through next-generation sequencing. J. Med. Virol..

[10] Frias-Staheli N., Giannakopoulos N.V., Kikkert M., Taylor S.L., Bridgen A., Paragas J., Richt J.A., Rowland R.R., Schmaljohn C.S., Lenschow D.J. (2007). Ovarian tumor domain-containing viral proteases evade ubiquitin- and ISG15-dependent innate immune responses. Cell Host Microbe.

[11] Akutsu M., Ye Y., Virdee S., Chin J.W., Komander D. (2011). Molecular basis for ubiquitin and ISG15 cross-reactivity in viral ovarian tumor domains. Proc. Natl. Acad. Sci. USA.

[12] Capodagli G.C., McKercher M.A., Baker E.A., Masters E.M., Brunzelle J.S., Pegan S.D. (2011). Structural analysis of a viral ovarian tumor domain protease from the Crimean-Congo hemorrhagic fever virus in complex with covalently bonded ubiquitin. J. Virol..

[13] James T.W., Frias-Staheli N., Bacik J.P., Macleod J.M.L., Khajehpour M., García-Sastre A., Mark B.L. (2011). Structural basis for the removal of ubiquitin and interferon-stimulated gene 15 by a viral ovarian tumor domain-containing protease. Proc. Natl. Acad. Sci. USA.

[14] van Kasteren P.B., Beugeling C., Ninaber D.K. (2012). Arterivirus and nairovirus ovarian tumor domain-containing deubiquitinases target activated RIG-I to control innate immune signaling. J. Virol..

[15] Scholte F.E.M., Spengler J.R., Welch S.R., Nichol S.T., Pegan S.D., Spiropoulou C.F., Bergeron É. (2017). Crimean-Congo hemorrhagic fever virus suppresses innate immune responses via a ubiquitin and ISG15 specific protease. Cell Rep..

[16] Devignot S., Kromer T., Mirazimi A., Weber F. (2020). ISG15 overexpression compensates the defect of Crimean-Congo hemorrhagic fever virus polymerase bearing a protease-inactive ovarian tumor domain. PLoS Negl. Trop. Dis..

[17] Kocabas F., Aslan G.S. (2015). Fluorometric CCHFV OTU protease assay with potent inhibitors. Virus Genes.

[18] Beckmann L., Götting D., Icker M., Rieger D., Schlegel P., Urban N., Schaefer M., Meiler J., Schoeder C.T., Tretbar M. (2025). Identification and optimization of a small molecule inhibitor of the ovarian tumor protease of the Crimean-Congo hemorrhagic fever virus. Bioorg Med. Chem..

[19] Scholte F.E.M., Hua B.L., Spengler J.R., Dzimianski J.V., Coleman-McCray J.D., Welch S.R., McMullan L.K., Nichol S.T., Pegan S.D., Spiropoulou C.F. (2019). Stable occupancy of the Crimean-Congo hemorrhagic fever virus-encoded deubiquitinase blocks viral infection. mBio.

[20] Scholte F.E.M., Spengler J.R., Welch S.R., Harmon J.R., Coleman-McCray J.D., Davies K.A., Pegan S.D., Montgomery J.M., Spiropoulou C.F., Bergeron É. (2024). Evaluation of two inoculation routes of an adenovirus-mediated viral protein inhibitor in a Crimean-Congo hemorrhagic fever mouse model. Virus Res..

[21] Hashim H.O., Omran A.M., Al-Hindy H.A.M., Abdulkareem Y.S., Abd Z.S., Rebat E.A., Hadi R.K. (2025). Repurposing FDA-approved drugs against the Crimean-Congo haemorrhagic fever virus: An in silico study. Pharmakeftiki.

[22] Kocabas F., Ergin E.K. (2016). Identification of small molecule binding pocket for inhibition of Crimean-Congo hemorrhagic fever virus OTU protease. Turk. J. Biol..

[23] Siyah P., Akgol S., Durdagi S., Kocabas F. (2021). Identification of first-in-class plasmodium OTU inhibitors with potent anti-malarial activity. Biochem. J..

[24] Kocabaş F., Uslu M. (2021). The current state of validated small molecules inhibiting SARS-CoV-2 non-structural proteins. Turk. J. Biol..

[25] Hashim H.O., Al-Hindy H.A.A.M., Razzaq M.R., Badr S.H., Ali Z.A., Abdulwahid T.R. (2025). Targeting the Crimean-Congo haemorrhagic fever virus with natural compounds: An in silico study. Pharmakeftiki.

[26] Hassam M., Rubina Zheng H., Moin S.T., Uddin R. (2026). Targeting Crimean–Congo hemorrhagic fever virus: Computational and MD-driven discovery of *Moringa oleifera* peptides against the RdRp-embedded OTU protease. Sci. Rep..

[27] Liu K., Li L., Liu Y., Wang X., Liu J., Li J., Deng F., Zhang R., Zhou Y., Hu Z. (2024). Discovery of baloxavir sodium as a novel anti-CCHFV inhibitor: Biological evaluation of in vitro and in vivo. Antivir. Res..

[28] O’Boyle N.M., Banck M., James C.A., Morley C., Vandermeersch T., Hutchison G.R. (2011). Open Babel: An open chemical toolbox. J. Cheminform..

[29] Diedrich K., Krause B., Berg O., Rarey M. (2023). PoseEdit: Enhanced ligand binding mode communication by interactive 2D diagrams. J. Comput.-Aided Mol. Des..

[30] Stierand K., Rarey M. (2007). From modeling to medicinal chemistry: Automatic generation of two-dimensional complex diagrams. ChemMedChem.

[31] Stierand K., Rarey M. (2010). Drawing the PDB: Protein−ligand complexes in two dimensions. ACS Med. Chem. Lett..

[32] Mammadova A., Mermer A., Kocabaş F. (2021). Screening of the small molecule library of Meinox enables the identification of anticancer compounds in pathologically distinct cancers. Turk. J. Biol..

[33] Yıldırım S., Kocabaş F., Mermer A. (2024). Development, synthesis and validation of improved c-Myc/Max inhibitors. J. Cell. Mol. Med..

